# Serious limitations of the QTL/Microarray approach for QTL gene discovery

**DOI:** 10.1186/1741-7007-8-96

**Published:** 2010-07-12

**Authors:** Ricardo A Verdugo, Charles R Farber, Craig H Warden, Juan F Medrano

**Affiliations:** 1 Department of Animal Science, University of California Davis. Davis, CA 95616, USA; 2 Departments of Medicine, Biochemistry and Molecular Genetics, and Center for Public Health Genomics, University of Virginia, Charlottesville, VA 22908, USA; 3 Departments of Pediatrics and Neurobiology, Physiology and Behavior, University of California Davis. Davis, CA 95616, USA; 4 The Jackson Laboratory, Bar Harbor, ME 04609, USA

## Abstract

**Background:**

It has been proposed that the use of gene expression microarrays in nonrecombinant parental or congenic strains can accelerate the process of isolating individual genes underlying quantitative trait loci (QTL). However, the effectiveness of this approach has not been assessed.

**Results:**

Thirty-seven studies that have implemented the QTL/microarray approach in rodents were reviewed. About 30% of studies showed enrichment for QTL candidates, mostly in comparisons between congenic and background strains. Three studies led to the identification of an underlying *QTL *gene. To complement the literature results, a microarray experiment was performed using three mouse congenic strains isolating the effects of at least 25 biometric QTL. Results show that genes in the congenic donor regions were preferentially selected. However, within donor regions, the distribution of differentially expressed genes was homogeneous once gene density was accounted for. Genes within identical-by-descent (IBD) regions were less likely to be differentially expressed in chromosome 2, but not in chromosomes 11 and 17. Furthermore, expression of *QTL *regulated in *cis *(*cis *eQTL) showed higher expression in the background genotype, which was partially explained by the presence of single nucleotide polymorphisms (SNP).

**Conclusions:**

The literature shows limited successes from the QTL/microarray approach to identify *QTL *genes. Our own results from microarray profiling of three congenic strains revealed a strong tendency to select *cis-*eQTL over *trans-*eQTL. IBD regions had little effect on rate of differential expression, and we provide several reasons why IBD should not be used to discard eQTL candidates. In addition, mismatch probes produced false *cis-*eQTL that could not be completely removed with the current strains genotypes and low probe density microarrays. The reviewed studies did not account for lack of coverage from the platforms used and therefore removed genes that were not tested. Together, our results explain the tendency to report QTL candidates as differentially expressed and indicate that the utility of the QTL/microarray as currently implemented is limited. Alternatives are proposed that make use of microarray data from multiple experiments to overcome the outlined limitations.

## Background

The study of genetics of quantitative traits has benefited from the availability of new technologies that generate massive information at the genomic and transcriptomic levels [1]. Microarray technology has been recognized as a powerful tool that could aid in the identification of the genes underlying quantitative trait loci (QTL; [2,3]). Microarray data can be analyzed within a QTL context following a genetical genomics (GG) approach [4]. This methodology considers gene expression values as a quantitative trait that can be mapped to chromosomal locations in a segregating population. Such genomic positions are called expression QTL (eQTL), which can be either *cis*- or *trans*-acting modifiers of gene expression, depending on whether they are located in the vicinity of or far from the measured gene, respectively. In practice, the validity of this distinction depends on the resolution of the QTL analysis, i.e., the density of the genetic map and the size of the segregating population [5]. Here we refer to QTL for phenotypes other than gene expression as pQTL to differentiate them from eQTL.

Some general ideas on eQTL can be drawn from relevant GG studies. Experiments in yeast [6], maize and mouse [7], and humans [8] found that most transcripts are affected by multiple loci, with each locus accounting for less than one third of parental expression differences. eQTL with the largest effects are located in close proximity to the target gene (within 10 Kb in yeast and within 1 Mb in mice and humans), which are referred to as proximal or *cis *eQTL [6,7]. However, most of the detected eQTL have been found to be *trans*-acting. The overall distribution of eQTL along the genome reveals the presence of "hot spots" with *trans-*eQTL for a large number of genes genome-wide [9]. These hubs of *trans-*eQTL do not necessarily represent transcription factors, but more likely represent a heterogeneous group of transcription regulators [10] or could simply be the result of unaccounted nongenetic correlation among transcripts [11]. When real, *trans*-eQTL hot spots can be used to identify gene modules under common genetic regulation [12]. Their detection, however, requires a larger sample size than typical GG studies provide; hence, they are missed in smaller-scale designs [9,13,14]. *cis-*eQTL are unique by some desirable properties, e.g., (1) known location of the causal gene and (2) their effect sizes are usually large and can be detected with smaller sample sizes. In general, *cis-*eQTL are regarded as strong quantitative trait gene (QTG) candidates when they are located under pQTL, their expression is correlated with the phenotype, and they tend to be located in regions not involved in identity-by-descent (IBD) relationships [15-17]. However, the cost of large-scale microarray profiling of a segregating population restricts the application of the GG approach. Therefore, experimental designs requiring a lower number of microarrays for the identification of *cis-*eQTL are desirable.

An alternative approach is defined in this paper as the QTL/microarray approach. This approach refers to the combined use of traditional QTL mapping and subsequent microarray profiling of nonrecombinant parental or congenic strains to reduce the number of candidate genes in QTL regions [18-20]. The methodology used in QTL/microarray studies, even though it varies among researchers, shares some common procedures that can be summarized as follows: (1) QTL mapping experiment and identification of genes located within confidence or support intervals for QTL, (2) a test of differential expression between parental strains (*Parental *design), (3) cross-reference list of positional candidates from step 1 and expression candidates from step 2, (4) hypothesis- or knowledge-driven filtering of the list of candidates, (5) independent confirmation of differential expression, and (6) experimental validation of causative genes. Step 3 sometimes compares gene expression on congenic and background strains (*Congenic *design) or between animals with extreme phenotypes from a segregating population (*Extremes *design). Step 5 often involves measuring expression of candidate genes by qRT-PCR in an independent set of samples, but can also involve Northern or Western blot analysis. Step 6 is usually part of a separate project that follows a QTL/microarray study.

The rationale behind QTL/microarray studies is that causative genes may have polymorphisms causing differences in their level of expression that translate into varying amounts of mRNA and ultimately varying amounts of functional proteins, leading to observable phenotypes. There are several mechanisms by which a QTG could change gene expression levels. A mutation in the binding site for a transcription factor may, for instance, decrease its binding affinity to the ligand, affecting the gene's transcription level [21]. Mutations in the transcription factors themselves could also affect recognition of their targets, thus also changing the gene's transcription level. Likewise, nonsense mutations in the coding sequence of a gene can decrease transcript levels by nonsense-mediated decay [22].

In contrast to the GG approach, transcript profiling of nonrecombinant animals does not allow QTL mapping of the expression levels and therefore cannot differentiate between *cis*- and *trans*-eQTL. However, co-localization of a differentially expressed gene and the pQTL can be tested, given that a physical, or genetic, map is available for the genes. This would be equivalent to a *cis-*eQTL/pQTL co-localization test for the genes under *cis *control. An approach for sorting out *cis*- from *trans*-eQTL in this experimental design consists of first isolating the genomic region with the pQTL in a congenic strain by backcrossing a donor strain to a recipient strain for multiple generations and then testing differential gene expression between the congenic and the recipient background strain. Depending on the size of the congenic strain, differentially expressed genes in the donor region are likely to be under *cis *regulation or alternatively by *trans *control from linked genes within the limits of the congenic interval. In contrast, differential expression of genes outside the donor region is expected to be regulated, directly or indirectly, by genes located within the congenic interval. Contaminating donor DNA in places outside the congenic donor region could produce false *trans*-eQTL. However, nonrecombinant individuals from an F_2 _cross between the congenic and background strains can be used to randomize the effect of contaminating regions.

The QTL/microarray approach is not exempt from issues that need to be addressed. A well-known problem associated with these studies is that DNA polymorphisms can affect the binding of microarray probes and significantly decreasing detectable signals. Such artifacts can produce an increase in false-positive results when genetically divergent individuals are compared with microarrays [23,24]. Presumably, however, this is not the only issue that may affect QTL/microarray results. Despite the wide use of the approach, no systematic evaluation of its performance has been reported.

In the current paper, a critical study of the QTL/microarray approach applied to the analysis of complex traits with clinical relevance in humans was performed. First, the literature was reviewed and a meta-analysis of rodent studies that have implemented this approach was conducted. Second, a microarray experiment with three mouse congenic strains was designed to test whether differential expression is associated with QTL peaks. The advantages and limitations of the QTL/microarray approach are discussed, and recommendations for the effective use of microarrays for the dissection of complex traits are provided.

## Results and Discussion

### Literature review

Thirty-seven published studies using the QTL/microarray approach in rats (Table 1) or mice (Table 2) were examined for key features of their experimental design. Most of the microarrays used were whole-genome arrays, including both cDNA, especially for older studies, and oligonucleotide arrays. Although the objective of using microarrays in most of these studies was similar, i.e., identification of differentially expressed genes under pQTL peaks, the methodologies used varied greatly. Particularly, no standards were followed in the statistical methodologies used for testing differential expression. Some studies do not report the methodology used at all [25,26]. Others report from one to three sequentially applied criteria to select lists of differentially expressed genes, which varied in nature and in statistical support. For instance, arbitrary thresholds by absolute intensity difference or fold changes (FC) [27-33], different combinations of FC and *t*-statistics [34-36], ranking by FC and selecting only the top 100 most significant genes [37], correlation to a hypothetical constant gene [38], and concordance in FC direction across experimental groups [39], among others (for the complete list studies with details about experimental design, see Additional file 1). Although applying multiple criteria can be attractive in terms of reducing the number of candidates, this is likely to reduce power in an unpredictable manner. This is further complicated when a criterion such as fold change (FC) is used, which has unknown significance level.

**Table 1:** Published studies analyzing parental strains using the QTL/Microarray approach in rat

	Phenotype	QTL Mapping		Microarray Experiment			References
				
		Design	Strains	Design	Tissues	Platform	
	***Targeted custom arrays***						
	Hypertension	Congenic	DS, LEW	Congenic	Kidney	Custom/ TIGR	[43]
	Hypertension	F2	DS, LEW	Parental	Kidney	RT-PCR	[31]

	***Whole-Genome arrays***						
•	Aerobic running capacity	Congenic	DS, DR	Parental	Kidney	RGU34	[34,95,96]
	Alcohol addiction	Congenic	iP, iNP	Congenic	NAC, FC, ABR, hippocampus, striatum	RG230v2	[97]
	Hypertension	F2	SBH/y, SBN/y	Parental	Kidney	RAE230	[41,98]
•	Hypertension	Congenic	DS, LEW	Congenic	Kidney	RGU34	[30]
•	Hypertension	Congenic	DS, DR	Congenic	Kidney	RGU34	[42,99]
•	Hypertension	Congenic	SHRSP, WKY	Congenic	Kidney	RGU34	[44]
	Hypertension	F2	DS, LEW	Parental	Kidney	RAE230	[32]
	Hypertension	F2	DS, LEW	Parental	Kidney, adrenal gland, liver, brain	RG230v2	[33]
•	Kidney stones	Congenic	GHS, WKY	Congenic	Kidney, SI, FOC	RAE230	[37]
	Metabolic syndrome	Congenic	SHR, WKY	Congenic	EWAT	Spotted	[27]
	Hypertension	Congenic	DS, DR	Parental	Kidney	GF 300	[40]

***Tissue abbreviations: ***ABR: amigdala brain region, EWAT: Epididymal white adipose tissue, FC: frontal cortex, FOC: femoral osteoclast cells, NAC: nucleus accumbens, SI: small intestine. ***Strain abbreviations: ***DS: Dahl salt-sensitive, DR: Dahl salt-resistant, GHS: genetic hypercalciuric stone-forming, iP: alcohol non-preferring, LEW: Lewis rats, P: alcohol preferring, SHR: spontaneously hypertensive rat, SHRSP: stroke-prone spontaneously hypertensive rat, SBH/y: Sabra salt-sensitive hypertensive, SBN/y: Sabra salt-resistant normotensive, WKY: Wistar Kyoto.

• Articles included in Table 3.

**Table 2:** Published studies analyzing parental strains using the QTL/Microarray approach in mouse

	Phenotype	QTL Mapping		Microarray Experiment			References
				
		Design	Strains	Design	Tissues	Platform	
	***Targeted custom arrays***						
	Arthritis	Congenic	RIIIS/J, B10.RIII	Congenic	Lymph nodes, spleen, paws	Custom	[100]
	Obesity	Congenic	SPRET/Ei, C57BL/6J	Parental	Brain, EWAT, GSM, liver	Nimblgen	[101]

	***Whole-Genome arrays***						
•	Alcohol addiction	RIL	HAFT, LAFT	Parental	Brain	MGU74A	[39,102]
•	Alcohol addiction	RIL	C57BL/6J, DBA/2J	Parental	NAC, PFC, VTA	U74Av2	[103]
	Alcohol addiction	Congenic	ISS.ILS.Lore, ISS	Parental	CR	MO430A, MOE430B	[38,104]
•	Anxiety	Knockout/congenic	B6.129-Il10-/-, C57BL/6J	Congenic	ABR	MOE430	[105]
	Anxiety	Database	-	Parental	PFC, VS, TL, PG, CR	Spotted	[106]
•	Arthritis	AIL	DNA/1J, FVB/N	Parental	Lynph nodes	MOE430A	[107]
	Arthritis	AIL	DNA/1J, FVB/N	Parental	Lynph nodes	U430A	[36]
•	EIAD	F2/RIL	DBA/1, C57BL/6J	Extremes	Lung	NIA15k	[52]
•	Gallstones	BC	C57L/J, AKR/J	Parental	Liver	Spotted	[108,109]
•	HSCP	RIL, BC, Congenic	C57BL/6J, DBA/2J	Parental	HSC	Filter	[49]
•	IBD	BC	B6-Il10^-/-^, C3H-Il10^-/-^	Congenic	Colon	U74Av2	[35,110]
	Lung injury response	BC	A/J, C57BL/6J	Parental	Lung	Spotted	[29]
•	Macronutrient intake	Congenic	C57BL/6J, CAST/EiJ	Congenic	Liver, hypothalamus	ABI	[103]
•	Obesity	Congenic	F-line, L-line	Congenic	Liver, BAT	Spotted	[111]
•	Obesity	BC	C57BL/6J, SPRET/Pt, SPRET/Ei	Extremes	EWAT	Spotted	[112]
•	Obesity	F2	DU6i, DBA/2	Parental	EWAT	Mu11k	[47]
•	Osteoporosis	Congenic	C57BL/6J, CAST/Ei	Congenic	Femur	Spotted	[28,113]
	Osteoporosis	RI	C57BL/6J, CAST/Ei	Parental	Kidney	Spotted	[26,45]
	Platelet count	F2	CBA/CaH, QSi5	Parental	Liver, kidney, spleen	Spotted/Compugen Mouse 22K	[25]
•	Pulmonary capacity	F2/BC	C3H/HeJ, JF1/Msf	Parental	Lung	Spotted/Operon	[114]
	TR	BC/Introgression	A/J, C57BL/6J	Parental	Liver, kidney, spleen	MG-430v2	[96,115]
•	Type I diabetes	Congenic	NOD, Idd3, Idd5, Idd3+5, Idd9, B10, B10.H2g7, B10.H2g7Idd3	Congenic	Thymus, Spleen	Affy.Eos.Custom	[116]

***Phenotype abbreviations: ***EIAD: Endotoxin-induced airway disease, HSCP: Hematopoietic stem cells proliferation, IBD: Inflammatory bowel disease, TR: Trypanosomiasis resistance. ***Tissue abbreviations: ***ABR: amigdala brain region, BAT: Brown adipose tissue, CR: cerebellum, GSM: gastrocnemius skeletal muscle, HSC: Hematopoietic stem cells, NAC: nucleus accumbens, PFC: Prefrontal cortex, PG: periaqueductal gray, TL: temporal lobe, VS: ventral striatum, VTA: ventral tegmental area. ***Strain abbreviations: ***HAFT: High Acute Functional Tolerance, ILS: Inbred Long Sleep, ISL: Inbred Short Sleep, LAFT: Low Acute Functional Tolerance. • Articles included in Table 3.

In some cases, the QTL/microarray design has led to the identification of a QTG. In rats, two studies were identified confirming the role of QTGs, i.e., Cd36 in insulin resistance [27] and Klk1 in hypertension [31]. Using a cDNA microarray, the Cd36 gene was selected as differentially expressed in adipose tissue between two divergent inbred strains of rats as well as between a congenic strain isolating a *QTL *on chromosome 4 and the background strain. This gene was located within the limits of the congenic donor region, but it was not mentioned whether other genes in the region were also differentially expressed [27]. This finding suggested Cd36 as a candidate associated with insulin resistance syndromes. Northern blots and sequencing revealed that the microarray probe targeting the 3' end of the mRNA molecule detected alternative splicing of the last exon and not differential expression. This was translated into absence of protein levels in plasma, and a transgenic model confirmed the effects of Cd36 on lipid levels in the blood [27]. It was not mentioned by Atiman *et al*. [27] how many genes were located within the congenic region, presumably because it was not known at the time of publication, when a physical map of the rat genome was not available. Unfortunately, the identity of the microsatellite markers defining the donor region was not provided, and it was not possible to calculate the size of the region. For this reason, the study by Aitman *et al*. [27] was not included in the meta-analysis in Table 3.

**Table 3:** Overrepresentation test for pQTL candidate genes in lists of differentially expressed genes in QTL/microarray experiments^a^

Phenotype	pQTL genes	Microarray Experiment		Selected Genes		Genome		Microarray		References
						
		Platform	Probes	GW	QTL	OR	P	OR	P	
***Rat***										
**Congenic**										
Hypertension	198	RGU34	26379	45	3	8.15	**0.007**	9.57	**0.005**	[44]
Hypertension	267	RGU34	26379	27	0	0.00	1.0	0.00	1.0	[30]
Hypertension	78	RGU34	26379	20	1	15.31	0.067	17.95	0.058	[42,99]
Kidney stones	551	RAE230	31042	50	16	19.27	**3.1e-14**	26.76	**2.2e-16**	[37]
**Parental**										
Aerobic running capacity	467	RGU34	26379	199	9	2.26	**0.024**	2.66	**0.009**	[34,95,96]

***Mouse***										
**Congenic**										
Anxiety	187	MOE430	45037	9	3	17.60	0.062	30.12	**0.037**	[105]
IBD	5541	U74Av2	12488	94	16	0.77	0.377	0.26	**2.6e-08**	[35,110]
Macronutrient intake	1230	ABI	32991	3101	185	1.35	**3.9e-04**	1.75	**1.4e-10**	[103]
Obesity	503	Spotted	14938	13	1	4.30	0.221	2.39	0.359	[111]
Osteoporosis	27	Spotted	8734	283	1	3.56	0.253	1.15	0.59	[28,113]
Type I diabetes	1294	Affy.Eos Custom	39000	170	17	2.17	**0.006**	3.27	**5.6e-05**	[116]
**Parental**										
Alcohol addiction	3251	MGU74Av2	12488	169	6	0.26	**1.4e-04**	0.10	**6.3e-15**	[39,102]
Alcohol addiction	4423	U74Av2	12488	996	151	0.88	0.18	0.30	**1.5e-49**	[48]
Arthritis	649	MOE430A	22000	1396	18	0.50	**0.002**	0.41	**3.9e-05**	[107]
Gallstones	416	Spotted	8734	57	0	0.00	1.0	0.00	0.114	[108,109]
HSCP	867	Filter	5184	200	11	1.72	0.105	0.28	**1.6e-06**	[49]
	5079	Mu11k	13069	77	14	0.93	0.886	0.35	**1.4e-04**	[47]
Pulmonary capacity	1261	Spotted/Operon	31775	933	1	0.02	**4.9e-19**	0.03	**2.0e-15**	[114]
**Extremes**										
EIAD	460	NIA15k	15000	30	0	5.59	**4.8e-12**	3.10	**9.3e-07**	[52]
Obesity	1610	Spotted	11000	50	5	1.71	0.23	0.65	0.427	[112]

***Phenotype abbreviations: ***EIAD: Endotoxin-induced airway disease, HSCP: Hematopoietic stem cells proliferation, IBD: Inflammatory bowel disease.

^a^Number of pQTL candidate genes was extracted from references or calculated from UCSC Genome Browser within the limits of the pQTL confidence intervals or congenic donor regions. Selected Genes columns indicate number of differentially expressed genes genome-wide (GW) or in candidate regions (pQTL). OR columns denote the ratio of odds, i.e. fraction of candidate genes in the selected and in the references sets. P-values (P) for over- (OR > 1) or under-representation (OR < 1) of QTL candidate genes in list of selected genes were calculated using the genome and microarray probes as reference (see methods). Significant p-values (P < 0.05) are shown in bold.

The QTL/microarray approach has been extensively used to study hypertension. A total of nine studies were identified that compared gene expression between rat strains that showed spontaneous difference for blood pressure [31-33,40,41] or between congenic and background strains created from these lines [30,42-44]. These studies have led to the direct identification of as many as 50 candidate genes for hypertension and to the confirmation of at least one causal gene, namely, *Klk1 *[31]. Instead of microarrays, Iwai *et al*. [31] used real-time polymerase chain reaction (PCR) to test 399 transcripts located within the confidence interval for the QTL position. It was reported that among 240 transcripts that were detected in kidney tissue, two were differentially expressed, i.e., *Klk1 *and *Ngfg*. From these, only *Klk1 *was confirmed by Western blot analysis [31]. The role of *Klk1 *as a QTG was confirmed by alleviation of hypertension symptoms through adenoviral transfer of human Kallikrein 1. Because expression profiling was restricted to the target region of the QTL, this study was not included in the overrepresentation test in Table 3.

Among the mouse studies reviewed, one QTG was identified using the QTL/microarray approach, i.e., *Alox15*. Klein *et al*. [26] generated a congenic strain to isolate a QTL for peak bone mineral density on mouse chromosome 11 that was previously identified by Klein *et al*. [45]. This was followed by measuring gene expression in kidney tissue using a whole genome high-density array (see Table 2). *Alox15 *was the only gene reported as being differentially expressed in the congenic region between the strains. This was confirmed by qRT-PCR in kidney and osteoblast cell cultures. Furthermore, the role of *Alox15 *was confirmed by a complementation test with an Alox15^-/- ^knockout mouse as well as two drugs that inhibit the protein product coded by this gene [26]. There are over 2500 genes known in the donor region for the congenic strain, so as in previous examples, after QTL mapping, microarray testing was the single step that reduced the number of candidate genes the most. Unfortunately, it was not reported how many genes were differentially expressed genome-wide, and therefore this study also had to be excluded from our tests in Table 3.

### Meta-analysis

Because of missing information, statistical testing could be performed on only 20 of the 37 studies compiled (Table 1 and Table 2). In addition, microarrays do not cover all genes in the genome [46]. To control for the differences in the level of genome coverage between platforms used in each study, the meta-analysis was performed using two reference sets: genes in the genome and probes in the microarray (see Methods). Filtering by differential expression, on average, selected 1.9% of pQTL candidates' genes (range from 0% to 15%; Table 3). Of 20 publications with sufficient information, 6 (30%) reported differentially expressed genes that were significantly enriched and 3 (15%) studies were underrepresented for genes within the pQTL or congenic region when compared with the whole genome (*P *< 0.05). When using probes in the array as a reference, 7 (35%) were overrepresented and 7 (35%) were underrepresented (Table 3). The four experiments that were underrepresented for candidates only when using genes in the microarray as reference were performed on microarrays with low genome coverage (13K, [47], 12.5K, [35,48]) or on filter arrays [49]. Because the number of pQTL candidates represented in the microarrays was not available, we used the total number of genes under QTL intervals as an approximation. Low genome coverage produced overestimation of the true number of tested candidates, explaining their apparent underrepresentation among differentially expressed genes in these platforms.

**Figure 1:**
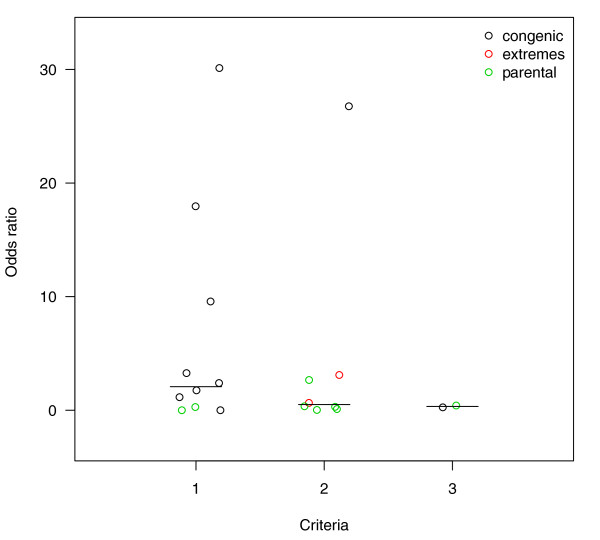
**Odd ratio for QTL candidates in list of differentially expressed genes**. Odds ratios for the enrichment of candidate genes in lists of differentially expressed genes versus all genes in the microarray (see Methods) is plotted versus number of selection criteria used by authors to reduce the number of selected genes. Studies are colored by type of microarray comparison: congenic vs. background strains (Congenic), parental inbred strains of the cross that was used for QTL mapping (Parental) or recombinant inbred lines (RIL). See Additional file 1 for details about the selection criteria used in each study.

The number of tests for differential expression applied in each study was used to question whether increasing the number of selection criteria increases the probability of selecting QTL candidate genes. No such trend was observed. In fact, the largest enrichments were seen in studies using a single selection criterion (Figure 1).

When divided by type of experiment, only one of eight *Parental *comparisons revealed enrichment for candidate genes. This is not unexpected, given that the test was performed not on QTG but only on positional candidates. Furthermore, inbred strains can present genome-wide genetic divergence that is not related to the specific phenotype under study. This limitation of the *Parental *design can be alleviated with the comparison of *Extremes*, where animals are specifically selected for the phenotype of interest and regions not harboring QTL are expected to segregate randomly relative to the phenotype. The *Extremes *design is the gene expression equivalent of mapping by allelic association where marker genotype frequencies are compared instead [50]. In both types of comparison, the degree to which differences between extremes are informative about an underlying association with the phenotype depends on the population and sample sizes, range of linkage disequilibrium (LD), and population history and structure; therefore, proper experimental design is required to avoid spurious associations [51]. Of two studies reviewed of this type, one presented significant enrichment. However, the one that showed significance used a modified design where the two extreme groups were composed of one parental strain and one recombinant inbred line [52]. Because of the confounding of two designs, i.e., *Parental *and *Extremes*, and its small sample size [only one recombinant inbred line (RIL) per extreme group], no further interpretation of this result is attempted. In contrast, the *Congenic *design was implemented in 11 studies with sufficient information for meta-analysis. Of these, five studies revealed enrichment for candidate genes. By design, only regions that are confirmed to harbor QTL have genetic divergence, and therefore enrichment of QTL candidates can be expected. However, multiple factors can potentially contribute to this trend. Polymorphic genomic regions are more likely to host pQTL for any trait and to generate allelic bias in probe binding for one strain versus the other. This situation can be expected in cases where the microarray has been designed for one of the strains that are being compared, or for a strain that is genetically more closely related to one of them. Allelic bias of probe binding will have a systematic effect on fluorescence intensity levels, which can be interpreted as differential gene expression. Since higher polymorphism rate is expected to increase both frequency of QTL as well as allelic bias of probe signals, the variables would be associated and the observed overrepresentation of pQTL candidates may result from such confounding effect. Both hypotheses are not mutually exclusive, since in reality QTL regions may be enriched for both polymorphisms that produce allelic bias as well as for functional polymorphisms that produce differential expression. However, it is important to assess the relative importance of these two factors in the apparent tendency for some QTL/microarray experiments to report pQTL candidates as differentially expressed.

Meta-analysis of results from the literature presents several limitations. Overrepresentation of pQTL candidates can be affected by a number of factors, such as publication bias for genes that are functional candidates or that are located near pQTL and inaccurate estimation of the gene coverage in microarrays. The number of candidate genes that were targeted by each microarray was largely unknown and the two reference sets used, i.e., total number of genes in the genome and number probes in the microarray, may not be optimum reference sets for overrepresentation tests. Furthermore, heterogeneity in quality of microarray annotation, definition of candidate region limits, and statistical procedures for data processing and differential expression testing limit our ability to investigate the specific causes of enrichment for pQTL candidates in their results. Therefore, an in-house microarray experiment was deemed necessary to specifically test for overrepresentation of candidate genes among differentially expressed genes. This gave us complete control over all these variables and allowed performing more specific tests that considered probe mismatches, IBD regions, and QTL location within congenic donor regions.

### Experiment using three congenic strains

**Figure 2:**
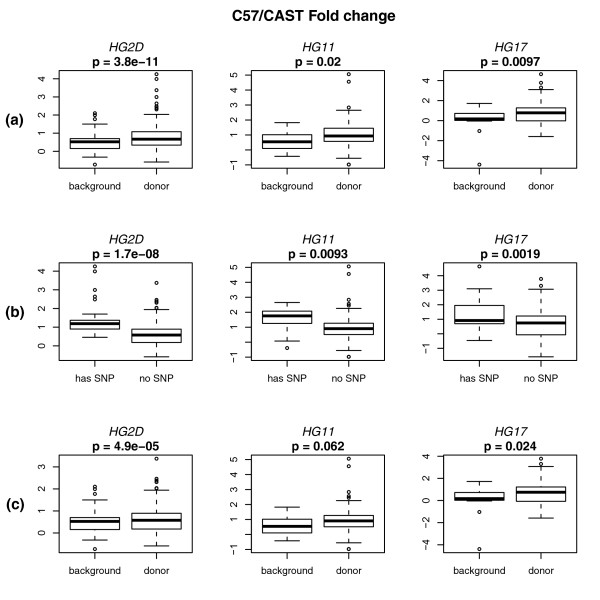
**Allelic bias in probe-level intensity**. Fold change for average intensity from samples of the C57 background genotype over the average from samples of the CAST congenic genotype is shown in log_2 _scale for congenic strains in chromosomes 2 (HG2D), 11 (HG11) and 17 (HG17). (a) Probes are separated by genomic location for genes within *donor *congenic region and genes in the *background *genome. (b) Probes are separated in to those that overlap at least one known SNP (*has SNP*) from those that do not (*no SNP*). (c) Plots in (a) after removing probes in donor region with SNP. *P *values for difference between groups are shown above each graph.

Differential expression between three CAST.C57.hg^-/- ^mouse congenic strains with their genotypic background controls C57.hg^-/- ^was tested on the Illumina Mouse-6 microarray with samples from brain, liver, and gonadal white adipose tissue (see Methods; data available at the NCBI GEO repository by accession GSE22042). This platform has coverage for 71.7% of 30,388 EntrezGenes in the mm9 genome assembly and for 75.5% of EntrezGenes in the donor region of the congenic strains. Differential expression analysis detected a total of 577, 110, and 109 genes (targeted by 682, 131, and 148 probes) genome-wide that were affected by allelic variants of genes in the congenic region of HG2D, HG11, and HG17, respectively. Of these, 124, 89, and 95 genes were located within the donor regions of those strains. Probes selected within the donor regions presented an allelic bias toward higher intensity of the C57 samples (Figure 2a). Since the reference mouse genome sequence used to design the microarray probes is C57, polymorphisms between this strain and CAST may have an effect on the binding affinity of microarray probes. A total of 410 probes overlapped at least one of the 289,541 known or imputed single nucleotide polymorphisms (SNP) within the limits of the donor regions (31 probes had two SNP, and one probe had three SNP). Of these, 209 probes detected a transcript in at least one tissue. This is in agreement with an observed bias toward higher intensity from C57 alleles in probes with known or imputed SNP (Figure 2b). However, allelic bias for genes in the donor region persisted even after these probes were removed (Figure 2c), suggesting that many SNP between C57 and CAST may still be unknown. However, it is also possible that the allelic bias is reflecting functional polymorphisms that can be detected in only one direction. For instance, insertions in the CAST genome would not be detected, whereas insertions in the C57 genome are detected as deletions in the CAST genome. Likewise, nonsense mutations causing RNA decay will only be apparent in CAST, since probes were designed for C57 mRNA molecules. On the basis of these findings, we recommend the use of high-density microarrays that target mRNA molecules in multiple locations, and custom probeset definitions can be designed to target only perfect matching sequence in the particular cross under study. The absence of this or alternative techniques [23] in all the reviewed papers leads us to conclude that such probe-binding artifacts also explain, at least in part, the increased frequency of candidate genes among reported differentially expressed genes.

Differential expression testing reduced the number of positional candidates from 1596, 1132, and 1347 to 124, 89, and 95 genes, that is, a reduction in 92.2%, 92.1%, and 92.9%, for HG2D, HG11, and HG17, respectively. However, these apparently high filtering rates result not only from lack of differential expression but also from lack of expression, removal of probe due to SNP, and lack of coverage of the microarray. Because untested genes cannot be discarded as candidates, genes that were targeted only by SNP-overlapping probes or not targeted at all in the microarray must be added back to the list of potential candidates. These represent 29%, 22.6%, and 33.2% of genes in the chromosomes 2, 11, and 17 donor regions, which were excluded from differential expression testing. The 124, 89, and 95 differentially expressed genes plus the 463, 256, and 447 genes that were not tested leaves a total of 587, 345, and 542 genes that would need further testing in each of the H2D, HG11, and HG17 congenic regions. The effective reduction of candidates by differential expression testing after adjustment was 63.2%, 69.5%, and 59.8%, respectively, i.e., 64% on average.

All differentially expressed genes located outside donor regions can be considered under *trans *regulation, in other words, *trans-*eQTL modulated. Genes regulated in *trans *were observed in almost every chromosome in the genome (Table 4). Differentially expressed genes within the congenic regions are candidates for *cis-*eQTL regulation and represented 21.5%, 80.1%, and 87.2% of selected genes, which is highly unlikely by chance considering that only 5.2%, 4%, and 4.2% of genes in the array are located in each of these regions (*P *= 6.38 × 10^-43^, *P *= 1.11 × 10^-104^, and *P *= 4.24 × 10^-117^) for chromosomes 2, 11, and 17, respectively. This high *cis-*eQTL enrichment was observed despite the fact that probes overlapping known SNP between CAST and C57 genomes were removed. Furthermore, some genes classified as *trans *regulated lay right at the ends of the congenic regions and are most likely *cis *regulated (Figure 2). This is expected because the limits for the donor regions used here represent minimum intervals from low-density genotyping [53], and the true limits may extend further than these intervals. We observed an approximate 3.1 to 1 ratio between *cis *and *trans *eQTL. It has been argued that selection acts distinctively on *cis *eQTL owing to quantitative effects, limited pleiotropy, and more exposure to selective pressure due to codominant effects versus a recessive mode of action characteristic of *trans *regulation [54]. Therefore, *cis *eQTL could have a predominant role in shaping genetic regulation of transcription [55]. However, empirical evidence compiled from GG studies in multiples species favors the view that *trans *eQTL are prevalent but show smaller effects than *cis *eQTL and can be missed at low sample sizes [6-8,56,57]. Although statistical issues related to multiple testing of *trans *eQTL and power to detect smaller effects makes it difficult to estimate the true ratio of *cis *versus *trans *regulatory loci [55], eQTL studies in yeast [58] and *Arabidopsis *[59] have shown that expression of most transcripts is most likely regulated by multiple loci, and a study in humans showed significant enrichment of interaction among multiple loci affecting gene expression [57]. Therefore, we hypothesize that the ratio observed here is due to the small experimental design with only four replicates per genotype and that the overrepresentation of candidates' genes in congenic QTL/microarray experiments may result from biased detection of *cis *eQTL as a consequence of low power [9,13,14].

**Table 4:** Number of differentially expressed genes outside congenic donor regions

Chromosome	Strain		
	HG2D	HG11	HG17
1	27	0	2
2	19	0	0
3	30	2	1
4	22	1	3
5	28	0	0
6	31	0	1
7	33	0	0
8	30	1	0
9	23	3	0
10	23	1	1
11	38	9	1
12	14	3	0
13	16	0	0
14	12	1	0
15	24	0	0
16	14	0	0
17	19	0	3
18	8	0	0
19	22	0	0
X	20	0	2

**Figure 3:**
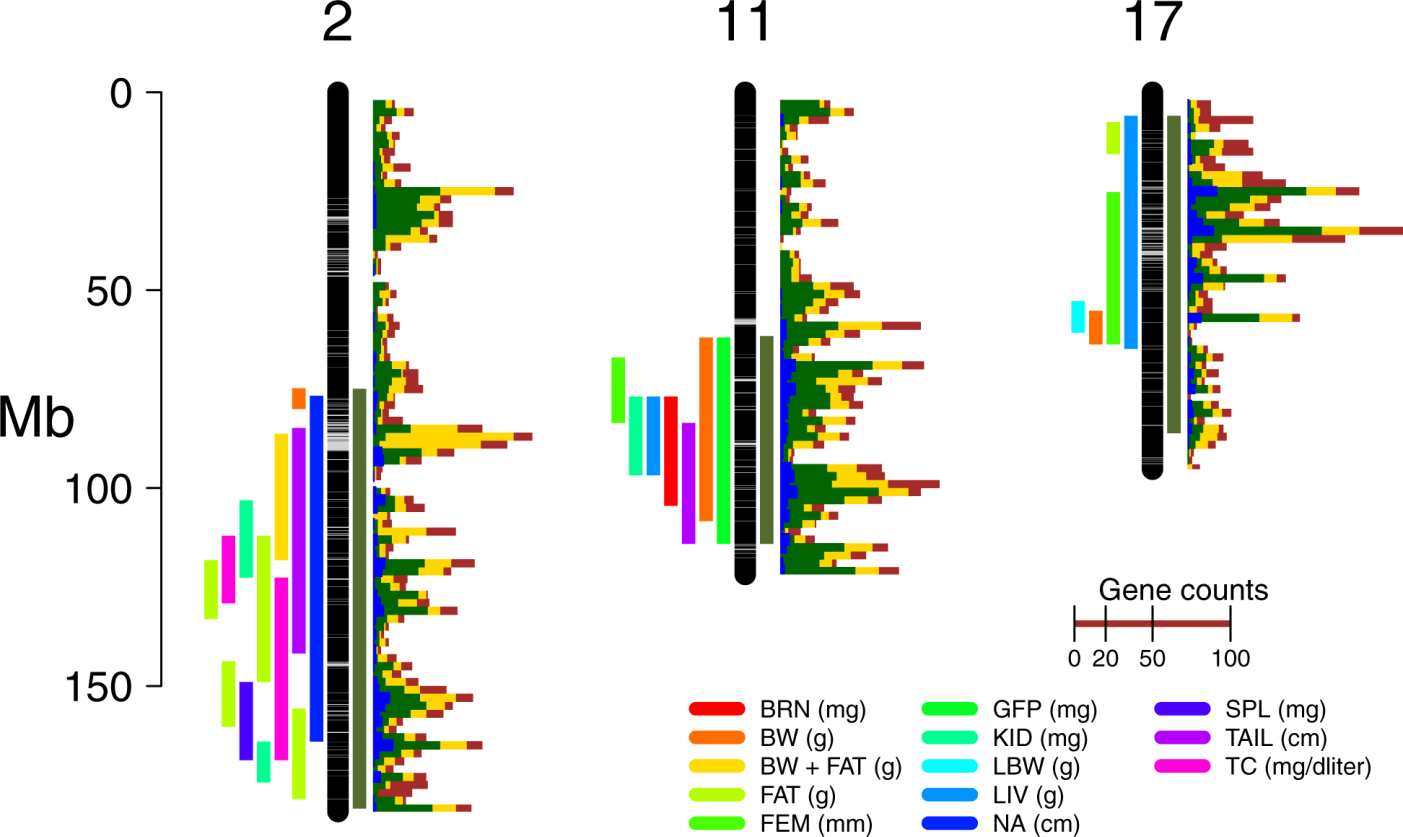
**Genes selected by differential expression on chromosomes 2, 11 and 17 from congenic microarray experiments**. Each chromosome is shown as a vertical black bar with the IBD regions shown as horizontal gray lines along the chromosome. The olive vertical line to the right of the chromosome represents the congenic donor region. The color bars to the left of the chromosome are the confidence intervals of the pQTL identified in the legend. A description of the phenotypes is in Additional file 2. The total number of genes in 2 Mbp bins is plotted in red bars to the right of the chromosomes. The number of genes in 2 Mbp bins along the chromosome is plotted with narrow horizontal bars to the right of each chromosome: brown, total number of genes; yellow, number of genes represented in the microarray; green, number of genes expressed in at least one tissue; blue, number of genes differentially expressed in at least one tissue. The scale labeled "Gene counts" represents the number of genes.

F2 offspring subcongenics from three congenic strains have been assayed for the same set of biometric measurements (Additional file 2) resulting in identification of at least 13, 7, and 5 QTL on chromosomes 2, 11, and 17 respectively [60,61] (Figure 3 and Additional file 3). The large number and overlap of QTL intervals would make it impossible to test for co-localization between differentially expressed genes and QTL. Instead, we tested whether the probability of differential expression was homogeneous along donor regions, conditioned on the number of expressed genes in bins of 2 Mb. A Fisher's exact test (see Methods) revealed no significant departure from homogeneity for chromosome 2 (*P *= 0.81), 11 (*P *= 0.52), or 17 (*P *= 0.67). Inspection of Figure 3 shows that the fraction of differentially expressed genes closely follows the distribution of genes in the donor regions (Figure 3). These results indicate that the distribution of selected genes within donor regions was mostly explained by the number of expressed genes and is not concentrated in any particular QTL region.

*cis *eQTL have been reported to be located preferentially in non-IBD regions [15], and their regulated genes have higher density of predicted SNP on transcription binding sites [21]. Therefore, multiple authors have proposed using the IBD status of genes to filter or prioritize *cis *eQTL candidates [15,20,62]. We tested whether genetic diversity within congenic regions was associated with differential expression. High genomic divergence between C57 and CAST resulted in only 14.7%, 6.4%, and 7.2% of the genes in donor regions of chromosomes 2, 11, and 17, respectively, to be located within IBD blocks. Using the IBD criteria discarded only 4, 3, and 9 genes from 124, 89, and 95 *cis *eQTL candidates in chromosomes 2, 11, and 17, respectively. Overrepresentation of differentially expressed genes within non-IBD blocks was observed only in chromosome 2 (*P *= 2.35 × 10^-6^) but not in chromosomes 11 (*P *= 0.35) or 17 (*P *= 0.54). Therefore, although *cis *eQTL candidates were preferentially located in non-IBD regions in agreement with Doss *et al*. [15], this was not significant once the probability of any gene to be located in IBD blocks is taken into account in chromosomes 11 and 17. This indicates that in this cross of highly divergent strains, enrichment of *cis *eQTLs in these two chromosomes was driven only by the overall higher rate of genes in those regions. This added to the limited number of genes that would be removed from the candidate list by the IBD criterion indicates that there would be no real gain in using this approach. Because we are inspecting only the chromosomes in one cross, we refrain from generalizing this conclusion to other cases. However, there are four main reason why using IBD to filter down lists of *cis *eQTL candidates should be done with care. In most cases, IBD is inferred from incomplete genotype data originated from resequencing [63], genotype imputation [64], or microarray genotyping for SNP discovered by the previous two methods [65]. Errors or lack of coverage from these methods could lead to imprecision in defining the size of IBD blocks that leads to filtering (or not) of genes that are actually non-IBD. Second, mutations that arose after the split of strain ancestors are missed from imputation techniques on the basis of a few highly divergent strains. Third, in the absence of evidence that the strain ancestors were homozygous at all loci, it is possible that modern strains have fixed different alleles of functional SNP that existed as heterozygous SNP in the strain ancestors. Fourth, enhancers in non-IBD regions may regulate expression of genes that are in IBD. We are aware of a least one case where this would have eliminated the causal gene if the IBD criterion were used. *Prcp *was identified as a candidate gene for obesity by subcongenic isolation and gene expression data from brain [66,67]. This gene is located in an IBD region between the donor strains BALB/cByJ and the background strain C57BL/6ByJ. However, in vitro assays with recombinant *PRCP *demonstrated that it has enzymatic activity to inactivate α-melanocyte-stimulating hormone (α-MSH_1-13_) by removing the C-terminal amino acid to produce α-MSH_1-12_. α-MSH_1-13_, a critical anorexigenic neuromodulator in the hypothalamus. A mouse model with a gene trap in *PRCP *confirmed effects of *PRCP *on obesity. In addition, inhibiting *PRCP *activity in vivo decreased food intake, confirming the role of *Prcp *in weight maintenance via control of active α-MSH_1-13 _levels [67]. Sequencing of this gene in the congenic revealed no SNP in the expressed sequence but only a promoter C→T transition that is hypothesized to affect the observed changes in gene expression and protein activity, food intake, and obesity phenotypes. This SNP was not known at that time and could not be inferred from the parental strains' ancestry. Therefore, identity by descent does not imply lack of DNA polymorphisms and, more important, of genetic differences that affect phenotypes.

In summary, differential expression testing in three congenic strains revealed expression signatures enriched for eQTL candidates, which resulted in hundreds of genes to be considered for further testing. Expression differences within the donor regions were distributed according to the overall distribution of genes in those regions and were affected by IBD blocks only on chromosome 2. High genetic divergence between C57 and CAST resulted in very limited number and size of IBD regions. Filtering by IBD would only discard 16 genes, which according to previous reports may well contain a causal variant. Intense phenotyping of F2 fine mapping populations revealed high genetic complexity, with multiple QTL, in each of these regions. The HG2D congenic includes multiple QTL for the same phenotype (body weight) with opposite genotype effects [61]. Furthermore, it is possible that many more QTL would be detected in these genomic regions if more phenotypes were collected. From the current data, it is impossible to distinguish between long-reaching linkage and pleiotropy or to infer causal relationships between QTL, transcripts, and organismal phenotypes. Phenotyping of large mapping populations would be necessary to break the association between these confounding effects.

The results from our literature review and the present experiment do not invalidate the use of microarrays for dissecting QTL. On the contrary, they stress the need for new approaches to make better use of these data. It has been shown that reanalyzing large repositories of microarray data can identify profiles of differential expression that are highly predictive of gene associations to human diseases [68,69]. By ranking genes by the ratio of experiments showing differential expression across many conditions, Chen *et al*. [68] were able to rediscover disease genes with 79% specificity and 37% sensitivity and proposed using this criterion to prioritize candidates resulting from GWAS associations. More recently, Gorlov *et al*. [69] found that the top genes by differential expression between normal and cancer tissue from human prostate are enriched for the same functional categories as the top candidate genes in GWAS and that strength of association in both tests was correlated. A similar approach could be employed in model organisms by reanalyzing results from a large number of microarray studies in inbred and congenic strains to increase both power and significance of genotype-phenotype associations. One could prioritize genes that are repeatedly differentially expressed between mouse genotypes that show similar phenotypic differences. Furthermore, such an approach would benefit from the availability of dense maps of SNP by using modern statistical tools to associate phenotypes to haplotypes produced by historical recombinations while accounting for genetic background and population structure [70]. Results from such analysis could be used to confirm and refine the position of eQTL from GG studies using F2 or backcrosses.

Using meta-analysis of large collections of microarray data to prioritize QTL candidates in rodents can present several advantages over similar approaches in humans. Linkage disequilibrium (LD) in humans can extend large distances; it is affected by population structure and history and can even reach across multiple chromosomes (see [71] and references therein). The situation is further complicated in case control designs where environmental factors significantly contribute to variation in gene expression [72]. In addition, technical factors such as time of preparation of samples for different populations have been proposed to explain some of the wide range of differences in gene expression observed between HapMap populations [55,73]. Environmental factors can be more tightly controlled in experimental populations, although differences between laboratories do have a significant effect on microarray results and must be considered [74]. Population structure is also present in laboratory mouse strains, and the contribution of different lines of ancestry is unequal across different regions of the genome [75], which would affect analyses of expression data across multiple strains. However, new populations can be designed to remove the effects of population structure. One population of note is the Collaborative Cross [76], which promises to greatly reduce LD and population structure (EJ Chesler, personal communication), and since it will be a panel of recombinant inbred strains, a large volume of phenotype and gene expression data is expected to accumulate over time. We think that these resources and the development of better methods for data analysis will greatly improve the success of using microarray data to dissect complex traits in rodents.

## Conclusions

A review of 37 studies from the literature that have applied the QTL/microarray showed that this approach is effective in reducing the list of candidate genes, with an average proportion of 1.9% candidate genes being differentially expressed across experiments. However, a meta-analysis of published results showed no significant overrepresentation of positional candidate genes among those selected in 70% of the studies. Most of the studies that did show enrichment of candidates were comparisons between congenic and background strains. Lack of standards in analytical methods for testing differential expression testing as well as a tendency to apply multiple criteria for probe selection was observed. Our analysis showed that no increase in enrichment is gained from this technique. In three reviewed studies, filtering by differential expression led to the identification of a QTG gene, where only a couple of genes were reported to be differentially expressed [26,27,77]. However, other studies with similar filtering ratios did not have such a favorable outcome (Table 3), and therefore luck played a role in determining the outcome in these studies.

By performing three independent congenic microarray experiments, we found high enrichment of genes within donor regions among differentially expressed genes. Within the limits of the donor regions, no clustering of differentially expressed genes to any particular pQTL region was observed, but a rather homogeneous distribution once overall gene density is accounted for. The high genetic divergence between C57 and CAST caused only ~6-15% of genes in the donor regions to fall in IBD blocks and genes within these blocks were less likely to be differentially expressed on chromosome 2 but not on chromosomes 11 or 17. On the basis of this and previous findings, IBD was not used to filter candidate genes. Furthermore, lack of genome coverage from the microarray used and removal of probes overlapping SNP excluded ~30% of positional candidates from differential expression testing. Overall, differential expression testing resulted in a reduction of the number of candidates by ~60-70%, leaving ~300-500 genes per donor region that need further testing. Therefore, our power to refine lists of candidate genes within donor regions from microarray data was rather poor. In addition, small sample size in our experiment restricted identification of *trans *eQTL. We expect that the same is true in the reviewed studies, contributing to the overrepresentation of pQTL candidates in some of them, resulting in a large number of candidates and making experimental validation impractical.

We showed that *cis *eQTL can be uninformative about QTG because they can show allelic bias toward higher expression of the reference genome. In our data, we found that this bias was partially explained by SNP on the binding region of probes, but that lack of full sequence of the CAST genome at the time of our analysis did not allow complete removal of this effect. Even with the availability of full CAST genome sequence, the limited number of probes in the platform used restricts the possibility of eliminating all probes matching SNP.

## Methods

### Collection of Microarray Studies

Compilation of studies using the QTL/microarray approach in mice and rats was performed by retrieving all citations that matched the Entrez query "(gene expression OR microarray) AND (QTL OR complex trait)" in PubMed [78], resulting in a list of 588 references. This list was manually curated by reading titles and abstracts and by keeping only studies that reported original results from using microarrays to identify candidates that cause differences in complex phenotypes in mouse and rat.

### Meta-analysis of QTL/Microarray studies

The analysis examined the overrepresentation or underrepresentation of genes within the pQTL or target regions that were selected in the reviewed studies. The information relevant for the objective of this analysis was (1) the number of genes under pQTL peaks, i.e., confidence limits for the position of pQTL, (2) number of genes in the genome being represented in the microarrays platform used, (3) number of differentially expressed genes, and (4) fraction of selected genes that are located within the confidence interval for the pQTL. In cases where microarray profiling was performed on congenic versus background strains, every gene located in the donor region of the congenic strain was considered a positional candidate.

Relevant information from all studies is shown in Tables 1 and 2 and was extracted as follows. The number of genes under a pQTL, if provided, was extracted from the text. Otherwise, it was estimated from the UCSC Genome Browser [79] by querying all known genes between the flanking markers for the pQTL confidence intervals; if flanking markers were not provided, physical or genetic confidence interval limits were obtained from the text or inferred from figures by visual inspection. Last, if no explicit confidence limits were plotted, a 1.5 drop in LOD from the pQTL peak was used. If confidence limits were only available as genetic positions, cM were transformed to Mbp using the results from a high-density SNP mapping experiment [80]. For experiments on congenic strains, flanking markers of the donor regions where used. NCBI mouse genome build 37 and rat build 3.4 were used to locate genetic markers in the physical genomic coordinates. The results of data collection are summarized in Table 3. The numbers of candidate genes in Table 3 are the totals for all pQTL considered in each study and should be regarded as approximate estimates of the real numbers.

Significance of over or under representation of pQTL candidate genes was assessed by a Fisher's exact test with the null hypothesis (*H*_*o*_):

where "candidate genes" refers to genes located within pQTL confidence intervals or donor congenic regions (depending on the experimental design of the microarray experiment), "genes in reference set" is the total number of genes considered, "selected candidate genes" is the number of genes differentially expressed in the candidate region, and "selected genes" is the total number of differentially expressed genes. In other words, under the null hypothesis, the detection of differentially expressed genes is assumed as a process of randomly sampling genes from a pool that includes genes within and outside the target region. Therefore, the fraction of target genes in the selected sample is expected to be equal to the fraction in the gene set that is used as a reference. The ratio of these two ratios is called the Ratio of Odds (OddsRatio). A Fisher's exact test for overrepresentation (OddsRatio > 1) or underrepresentation (OddsRatio < 1) of candidate genes in the list of differentially expressed genes was performed and *P *values were calculated from the hypergeometric distribution by using the *fisher.test *function in R. Two overrepresentation tests were performed by either using the total number of genes in the genome or the number of probes in the microarray. The number of genes in the genome was obtained from the assembly statistics at the Ensembl genome browser [81,82] by adding counts for "Known protein-coding," "Projected protein-coding," and "Novel protein-coding" gene categories (26,404 *M. musculus *genes, Build 37; 22,503 *R. norvegicus *genes, Build 3.4). The second test, using number of probes in the microarray, is intended to control for the effect of different levels of genome coverage by microarrays. An ideal reference would only consider genes that are included in the microarrays. However, because of nonexistent or obsolete annotations for some platforms, this information is not always known and we used the total number of probesets as an approximation.

### Experiment using three congenic strains

Three congenic mouse strains were profiled with microarrays and were analyzed using a QTL/microarray approach to identify candidate genes that regulate obesity traits: HG2D (HG.CAST-(D2Mit329-D2Mit457)), HG11 (HG.CAST-(D11Mit260-D11Mit255, MGI reference: 3771218), and HG17 (HG.CAST-(D17Mit196-D17Mit190); MGI reference: 3771215) [53]. All these congenic strains isolate CAST/EiJ (CAST) alleles in a C57BL/6J^*hg/hg *^(C57) background and bare the *hg *deletion in the *high growth *locus on chromosome 10 [83,84]. Animals for microarray profiling were generated from an F_2 _intercross between congenic males and C57BL/6J control females. Mice were weaned at 3 weeks old, housed in age- and sex-matching cages with five or fewer animals per cage. All animals were fed a standard Purina Formulab Chow 5008 diet and killed at 9 weeks old. Nonrecombinant animals for the congenic region that were homozygous for congenic or background alleles and *hg/hg *for the *high growth *locus were selected. Brain, liver, and gonadal white adipose tissues were collected from four biological replicates, snap frozen in liquid nitrogen, and stored at -80°C. All samples were obtained from males except for adipose tissue in HG2D. All mouse protocols followed the guidelines of the American Association for Accreditation of Laboratory Animal Care [85].

RNA was purified, prepared, and hybridized using manufacturers' protocols. Briefly, total RNA was extracted with TRIzol reagent (Invitrogen, Carlsbad, CA), DNase treated (TurboDNA, Ambion Inc., Austin, TX), and cDNA was generated by reverse transcription using Total Prep RNA Amplification Kit (Illumina Inc., San Diego, CA). cDNA was labeled with Biotin-16-UTP and hybridized to Mouse-6 V1 Illumina BeadArrays at the Gene Expression Core facility of the University of California Davis Genome Center (Davis, CA). BeadArrays were scanned and features were extracted using BeadStudio V. 1.5.1.3 (Illumina Inc., San Diego, CA). Local background correction was done at scanning using default values. Bead level data was summarized by removing outliers greater than 3 median absolute deviations (MADs) from the median and by calculating the mean and variance of the remaining beads. Summarized intensity values were imported into the R 2.9.2 language/environment for normalization and analysis [86].

The probes in Mouse-6 array were gene-annotated in-house to ensure current coverage of the mouse genome. Probe sequences were aligned to the mm9 mouse genome (Genome Build 37) obtained from the UCSC Genome Browser [87]. Sequence alignment was performed with stand-alone BLAT [88]. Probes mapping to multiple locations or with gaps larger than 10 Kb were not annotated. The resulting alignments were overlaid to, in this order, NCBI's RefSeqs [89], mouse mRNA, and human proteins mapped to the mouse genome by UCSC [79]. Intensity values were normalized with a quantiles the *affy *package from Bioconductor [90]. Probes were filtered by present calls (*P *< 0.01) in four or more samples from any given tissue. A total of 72 samples (3 strains × 2 genotypes × 3 tissue × 4 replicates) were hybridized to 12 Mouse-6 chips. Differential expression was tested by fitting a cell-means linear model after background correction and normalization of single-sample intensity values,

where *y*_*ij *_is the base 2 log transformed gene expression, *μ *is the overall mean, *μ*_*i *_is the effect of experimental group *k *(*k *= 1,...,18), and *ε*_*ij *_is the residual effect (*j *= 1,...,4). Experimental group was defined as the combination of strain, genotype within strain, and tissue. For each strain, there are six experimental groups: B.B, B.C, F.B, F.C, L.B, and L.C, where the first letter indicates tissue (B = brain, F = fat, and L = liver) and the second letter is genotype (B = C57 and C = CAST). Differential expression by genotype was tested by the following contrasts: L.Geno = L.C-L.B, F.Geno = F.C-F.B, and B.Geno = B.C-B.B. Model fitting and contrasts testing was done with the R/Maanova software [91]. *F *values were calculated with James-Stein shrinkage estimates of the error variance [92]. *P *values were calculated by 1000 permutations of sample labels and by pooling permutation *F *values across probes. Multiple comparison error was controlled by a false discovery rate (FDR) transformation [93]. Probe with a FDR <10% for any tissue were selected.

Over- or underrepresentation of positional candidates in the set of differentially expressed genes was tested by Fisher's exact test as described above for meta-analysis of literature results. For the test of homogeneity or rations of differentially expressed genes along donor regions, *P *values were estimated by sampling 2 million Monte Carlo simulations of the possible contingency tables using the *fisher.test *R function. Probes targeting transcripts associated to the same EntrezGenes were grouped so that overrepresentation tests counted genes as selected if any of their transcripts were found differentially expressed. Probes within the congenic regions that overlapped at least one SNP from between C57 and CAST were not considered [64]. Significance was determined from a Fisher's exact test (α = 0.05) as described above. Every gene within the limits of the donor regions of the congenic strains was considered a candidate. A similar test was done for genes from non-IBD regions that were selected as differentially expressed. IBD blocks for chromosomes 2, 11, and 17 were inferred from a database of imputed genotypes [64] and downloaded from the CGD Strain Comparison web tool [94]. IBD regions were identified as contiguous blocks of 10 Kbp with 10 or more uninformative SNP between the C57 and CAST strains. Genes were considered in IBD regions if both the transcription start and end sites were located within IBD block boundaries.

## Authors' contributions

RV did the literature review, designed the microarray experiment with the HG17 congenic strain, analyzed the data and drafted the manuscript. CRF designed and performed the mouse crosses and microarray experiments with the HG2D and HG11 congenic strains. CHW oversaw the design of the study and helped to draft the manuscript. JFM conceived the study and participated in its design and coordination and helped to draft the manuscript. All authors read and approved the final manuscript.

## Supplementary Material

Additional file 1 **Metadata from QTL/Microarray Studies in Rat and Mouse**. Table with one row per each of 37 reviewed studies implementing the QTL/Microarray approach in rodents. Information about experimental design and results was collected. Additional sheets contain description of acronyms and the full list of references. Click here for file

Additional file 2 **List of phenotypes measured in three congenic strains HG2D, HG11, HG17**. The file contains a list, symbol and units of measurement for 16 phenotypes measured in the mice. Click here for file

Additional file 3 **QTL located within the limits of the donor regions for the HG2D, HG11, and HG17 congenic strains**. The file contains a table with interval limits representing a non-redundant set of QTL from the cited references at the highest resolution currently known. Click here for file

## References

[B1] Mackay TFC Stone EA Ayroles JF The genetics of quantitative traits: challenges and prospects Nat Rev Genet 2009 10 8 565 577 10.1038/nrg2612 19584810

[B2] Pomp D Allan MF Wesolowski SR Quantitative genomics: Exploring the genetic architecture of complex trait predisposition J Anim Sci 2004 82 13_suppl E300 E312 15471811 10.2527/2004.8213_supplE300x

[B3] Farrall M Quantitative genetic variation: a post-modern view Hum Mol Genet 2004 13 Spec No 1 R1 7 10.1093/hmg/ddh084 14962979

[B4] Jansen RC Nap JP Genetical genomics: the added value from segregation Trends Genet 2001 17 7 388 391 10.1016/S0168-9525(01)02310-1 11418218

[B5] McClurg P Janes J Wu C Delano DL Walker JR Batalov S Takahashi JS Shimomura K Kohsaka A Bass J Wiltshire T Su AI Genomewide Association Analysis in Diverse Inbred Mice: Power and Population Structure Genetics 2007 176 1 675 683 10.1534/genetics.106.066241 17409088 PMC1893038

[B6] Brem RB Yvert G Clinton R Kruglyak L Genetic dissection of transcriptional regulation in budding yeast Science 2002 296 5568 752 755 10.1126/science.1069516 11923494

[B7] Schadt EE Monks SA Drake TA Lusis AJ Che N Colinayo V Ruff TG Milligan SB Lamb JR Cavet G Linsley PS Mao M Stoughton RB Friend SH Genetics of gene expression surveyed in maize, mouse and man Nature 2003 422 6929 297 302 10.1038/nature01434 12646919

[B8] Morley M Molony CM Weber TM Devlin JL Ewens KG Spielman RS Cheung VG Genetic analysis of genome-wide variation in human gene expression Nature 2004 430 7001 743 747 10.1038/nature02797 15269782 PMC2966974

[B9] de Koning DJ Haley CS Genetical genomics in humans and model organisms Trends Genet 2005 21 7 377 381 10.1016/j.tig.2005.05.004 15908034

[B10] Yvert G Brem RB Whittle J Akey JM Foss E Smith EN Mackelprang R Kruglyak L Trans-acting regulatory variation in Saccharomyces cerevisiae and the role of transcription factors Nat Genet 2003 35 1 57 64 10.1038/ng1222 12897782

[B11] Perez-Enciso M In silico study of transcriptome genetic variation in outbred populations Genetics 2004 166 1 547 554 10.1534/genetics.166.1.547 15020443 PMC1470684

[B12] Ghazalpour A Doss S Zhang B Wang S Plaisier C Castellanos R Brozell A Schadt EE Drake TA Lusis AJ Horvath S Integrating genetic and network analysis to characterize genes related to mouse weight PLoS Genet 2006 2 8 e130 10.1371/journal.pgen.0020130 16934000 PMC1550283

[B13] de Koning DJ Carlborg O Haley CS The genetic dissection of immune response using gene-expression studies and genome mapping Vet Immunol Immunopathol 2005 105 (3 4):343-352 10.1016/j.vetimm.2005.02.007 15808311

[B14] Haley C de Koning DJ Genetical genomics in livestock: potentials and pitfalls Animal Genet 2006 37 Suppl 1 10 12 10.1111/j.1365-2052.2006.01470.x 16886996

[B15] Doss S Schadt EE Drake TA Lusis AJ Cis-acting expression quantitative trait loci in mice Genome Res 2005 15 5 681 691 10.1101/gr.3216905 15837804 PMC1088296

[B16] Bao L Peirce JL Zhou M Li H Goldowitz D Williams RW Lu L Cui Y An integrative genomics strategy for systematic characterization of genetic loci modulating phenotypes Hum Mol Genet 2007 16 11 1381 1390 10.1093/hmg/ddm089 17428815

[B17] Tsai J Sultana R Lee Y Pertea G Karamycheva S Antonescu V Cho J Parvizi B Cheung F Quackenbush J RESOURCERER: a database for annotating and linking microarray resources within and across species Genome Biol 2001 2 11 SOFTWARE0002 10.1186/gb-2001-2-11-software0002 16173164 PMC138985

[B18] Matthews DB Bhave SV Belknap JK Brittingham C Chesler EJ Hitzemann RJ Hoffmann PL Lu L McWeeney S Miles MF Tabakoff B Williams RW Complex genetics of interactions of alcohol and CNS function and behavior Alcohol Clin Exp Res 2005 29 9 1706 1719 10.1097/01.alc.0000179209.44407.df 16205371

[B19] Arbilly M Pisante A Devor M Darvasi A An integrative approach for the identification of quantitative trait loci Animal Genet 2006 37 Suppl 1 7 9 10.1111/j.1365-2052.2006.01472.x 16886995

[B20] Drake TA Schadt EE Lusis AJ Integrating genetic and gene expression data: application to cardiovascular and metabolic traits in mice Mamm Genome 2006 17 6 466 479 10.1007/s00335-005-0175-z 16783628 PMC2679634

[B21] GuhaThakurta D Xie T Anand M Edwards SW Li G Wang SS Schadt EE Cis-regulatory variations: a study of SNPs around genes showing cis-linkage in segregating mouse populations BMC Genomics 2006 7 235 10.1186/1471-2164-7-235 16978413 PMC1618400

[B22] Chang YF Imam JS Wilkinson MF The nonsense-mediated decay RNA surveillance pathway Annu Rev Biochem 2007 76 51 74 10.1146/annurev.biochem.76.050106.093909 17352659

[B23] Alberts R Terpstra P Bystrykh LV de Haan G Jansen RC A statistical multiprobe model for analyzing cis and trans genes in genetical genomics experiments with short-oligonucleotide arrays Genetics 2005 171 3 1437 1439 10.1534/genetics.105.045930 16079228 PMC1456841

[B24] Alberts R Terpstra P Li Y Breitling R Nap JP Jansen RC Sequence polymorphisms cause many false cis eQTLs PLoS ONE 2007 2 7 e622 10.1371/journal.pone.0000622 17637838 PMC1906859

[B25] Cheung CC Martin IC Zenger KR Donald JA Thomson PC Moran C Buckley MF Quantitative trait loci for steady-state platelet count in mice Mamm Genome 2004 15 10 784 797 10.1007/s00335-004-2408-y 15520881

[B26] Klein RF Allard J Avnur Z Nikolcheva T Rotstein D Carlos AS Shea M Waters RV Belknap JK Peltz G Orwoll ES Regulation of bone mass in mice by the lipoxygenase gene Alox15 Science 2004 303 5655 229 232 10.1126/science.1090985 14716014

[B27] Aitman TJ Glazier AM Wallace CA Cooper LD Norsworthy PJ Wahid FN Al-Majali KM Trembling PM Mann CJ Shoulders CC Graf D St Lezin E Kurtz TW Kren V Pravenec M Ibrahimi A Abumrad NA Stanton LW Scott J Identification of Cd36 (Fat) as an insulin-resistance gene causing defective fatty acid and glucose metabolism in hypertensive rats Nat Genet 1999 21 1 76 83 10.1038/5013 9916795

[B28] Gu W Li X Lau KH Edderkaoui B Donahae LR Rosen CJ Beamer WG Shultz KL Srivastava A Mohan S Baylink DJ Gene expression between a congenic strain that contains a quantitative trait locus of high bone density from CAST/EiJ and its wild-type strain C57BL/6J Funct Integr Genomics 2002 1 6 375 386 10.1007/s10142-001-0042-2 11957112

[B29] Prows DR McDowell SA Aronow BJ Leikauf GD Genetic susceptibility to nickel-induced acute lung injury Chemosphere 2003 51 10 1139 1148 10.1016/S0045-6535(02)00710-5 12718980

[B30] Moujahidine M Lambert R Dutil J Palijan A Sivo Z Ariyarajah A Deng AY Combining congenic coverage with gene profiling in search of candidates for blood pressure quantitative trait loci in Dahl rats Hypertens Res 2004 27 3 203 212 10.1291/hypres.27.203 15080379

[B31] Iwai N Yasui N Naraba H Tago N Yamawaki H Sumiya H Klk1 as one of the genes contributing to hypertension in Dahl salt-sensitive rat Hypertension 2005 45 5 947 953 10.1161/01.HYP.0000161969.65767.0d 15809361

[B32] Yasui N Kajimoto K Sumiya T Okuda T Iwai N The monocyte chemotactic protein-1 gene may contribute to hypertension in Dahl salt-sensitive rats Hypertens Res 2007 30 2 185 193 10.1291/hypres.30.185 17460389

[B33] Kajimoto K Hiura Y Sumiya T Yasui N Okuda T Iwai N Exclusion of the catechol-o-methyltransferase gene from genes contributing to salt-sensitive hypertension in dahl salt-sensitive rats Hypertens Res 2007 30 5 459 467 10.1291/hypres.30.459 17587758

[B34] Lee SJ Ways JA Barbato JC Essig D Pettee K DeRaedt SJ Yang S Weaver DA Koch LG Cicila GT Gene expression profiling of the left ventricles in a rat model of intrinsic aerobic running capacity Physiol Genomics 2005 23 1 62 71 10.1152/physiolgenomics.00251.2004 16033863

[B35] de Buhr MF Mahler M Geffers R Hansen W Westendorf AM Lauber J Buer J Schlegelberger B Hedrich HJ Bleich A Cd14, Gbp1, and Pla2g2a: three major candidate genes for experimental IBD identified by combining QTL and microarray analyses Physiol Genomics 2006 25 3 426 434 10.1152/physiolgenomics.00022.2005 16705022

[B36] Yu X Bauer K Koczan D Thiesen HJ Ibrahim SM Combining global genome and transcriptome approaches to identify the candidate genes of small-effect quantitative trait loci in collagen-induced arthritis Arthritis Res Ther 2007 9 1 R3 10.1186/ar2108 17244351 PMC1860061

[B37] Hoopes RR Middleton FA Sen S Hueber PA Reid R Bushinsky DA Scheinman SJ Isolation and confirmation of a calcium excretion quantitative trait locus on chromosome 1 in genetic hypercalciuric stone-forming congenic rats J Am Soc Nephrol 2006 17 5 1292 1304 10.1681/ASN.2005080828 16611718

[B38] MacLaren EJ Bennett B Johnson TE Sikela JM Expression profiling identifies novel candidate genes for ethanol sensitivity QTLs Mamm Genome 2006 17 2 147 156 10.1007/s00335-005-0065-4 16465594 PMC2677977

[B39] Tabakoff B Bhave SV Hoffman PL Selective breeding, quantitative trait locus analysis, and gene arrays identify candidate genes for complex drug-related behaviors J Neurosci 2003 23 11 4491 4498 12805289 10.1523/JNEUROSCI.23-11-04491.2003 PMC6740804

[B40] Lee SJ Liu J Qi N Guarnera RA Lee SY Cicila GT Use of a panel of congenic strains to evaluate differentially expressed genes as candidate genes for blood pressure quantitative trait loci Hypertens Res 2003 26 1 75 87 10.1291/hypres.26.75 12661916

[B41] Yagil C Hubner N Monti J Schulz H Sapojnikov M Luft FC Ganten D Yagil Y Identification of hypertension-related genes through an integrated genomic-transcriptomic approach Circ Res 2005 96 6 617 625 10.1161/01.RES.0000160556.52369.61 15731461

[B42] Garrett MR Meng H Rapp JP Joe B Locating a blood pressure quantitative trait locus within 117 kb on the rat genome: substitution mapping and renal expression analysis Hypertension 2005 45 3 451 459 10.1161/01.HYP.0000154678.64340.7f 15655120

[B43] Joe B Letwin NE Garrett MR Dhindaw S Frank B Sultana R Verratti K Rapp JP Lee NH Transcriptional profiling with a blood pressure QTL interval-specific oligonucleotide array Physiol Genomics 2005 23 3 318 326 10.1152/physiolgenomics.00164.2004 16204469

[B44] McBride MW Carr FJ Graham D Anderson NH Clark JS Lee WK Charchar FJ Brosnan MJ Dominiczak AF Microarray analysis of rat chromosome 2 congenic strains Hypertension 2003 41 3 Pt 2 847 853 10.1161/01.HYP.0000047103.07205.03 12624007

[B45] Klein OF Carlos AS Vartanian KA Chambers VK Turner EJ Phillips TJ Belknap JK Orwoll ES Confirmation and fine mapping of chromosomal regions influencing peak bone mass in mice J Bone Miner Res 2001 16 11 1953 1961 10.1359/jbmr.2001.16.11.1953 11697791

[B46] Verdugo RA Medrano JF Comparison of gene coverage of mouse oligonucleotide microarray platforms BMC Genomics 2006 7 58 10.1186/1471-2164-7-58 16551360 PMC1440853

[B47] Aksu S Koczan D Renne U Thiesen HJ Brockmann GA Differentially expressed genes in adipose tissues of high body weight-selected (obese) and unselected (lean) mouse lines J Appl Genet 2007 48 2 133 143 17495347 10.1007/BF03194671

[B48] Kerns RT Ravindranathan A Hassan S Cage MP York T Sikela JM Williams RW Miles MF Ethanol-responsive brain region expression networks: implications for behavioral responses to acute ethanol in DBA/2J versus C57BL/6J mice J Neurosci 2005 25 9 2255 2266 10.1523/JNEUROSCI.4372-04.2005 15745951 PMC6726093

[B49] De Haan G Bystrykh LV Weersing E Dontje B Geiger H Ivanova N Lemischka IR Vellenga E Van Zant G A genetic and genomic analysis identifies a cluster of genes associated with hematopoietic cell turnover Blood 2002 100 6 2056 2062 10.1182/blood-2002-03-0808 12200366

[B50] Collins A Morton NE Mapping a disease locus by allelic association Proceedings of the National Academy of Sciences of the United States of America 1998 95 4 1741 1745 10.1073/pnas.95.4.1741 9465087 PMC19174

[B51] Cardon LR Bell JI Association study designs for complex diseases Nat Rev Genet 2001 2 2 91 99 10.1038/35052543 11253062

[B52] Cook DN Wang S Wang Y Howles GP Whitehead GS Berman KG Church TD Frank BC Gaspard RM Yu Y Quackenbush J Schwartz DA Genetic regulation of endotoxin-induced airway disease Genomics 2004 83 6 961 969 10.1016/j.ygeno.2003.12.008 15177550

[B53] Farber CR Corva PM Medrano JF Genome-wide isolation of growth and obesity QTL using mouse speed congenic strains BMC Genomics 2006 7 1 102 10.1186/1471-2164-7-102 16670015 PMC1482699

[B54] Wray GA The evolutionary significance of *cis*-regulatory mutations Nat Rev Genet 2007 8 3 206 216 10.1038/nrg2063 17304246

[B55] Gilad Y Rifkin SA Pritchard JK Revealing the architecture of gene regulation: the promise of eQTL studies Trends Genet 2008 24 8 408 415 10.1016/j.tig.2008.06.001 18597885 PMC2583071

[B56] Hubner N Wallace CA Zimdahl H Petretto E Schulz H Maciver F Mueller M Hummel O Monti J Zidek V Musilova A Kren V Causton H Game L Born G Schmidt S Müller A Cook SA Kurtz TW Whittaker J Pravenec M Aitman TJ Integrated transcriptional profiling and linkage analysis for identification of genes underlying disease Nat Genet 2005 37 3 243 253 10.1038/ng1522 15711544

[B57] Dixon AL Liang L Moffatt MF Chen W Heath S Wong KC Taylor J Burnett E Gut I Farrall M Lathrop GM Abecasis GR Cookson WO A genome-wide association study of global gene expression Nat Genet 2007 39 10 1202 1207 10.1038/ng2109 17873877

[B58] Brem RB Kruglyak L The landscape of genetic complexity across 5,700 gene expression traits in yeast Proc Natl Acad Sci USA 2005 102 5 1572 1577 10.1073/pnas.0408709102 15659551 PMC547855

[B59] West MA Kim K Kliebenstein DJ van Leeuwen H Michelmore RW Doerge RW St Clair DA Global eQTL mapping reveals the complex genetic architecture of transcript-level variation in Arabidopsis Genetics 2007 175 3 1441 1450 10.1534/genetics.106.064972 17179097 PMC1840073

[B60] Farber CR Medrano JF Fine mapping reveals sex bias in quantitative trait loci affecting growth, skeletal size and obesity-related traits on mouse chromosomes 2 and 11 Genetics 2007 175 1 349 360 10.1534/genetics.106.063693 17110492 PMC1775020

[B61] Farber CR Medrano JF Dissection of a genetically complex cluster of growth and obesity QTLs on mouse chromosome 2 using subcongenic intercrosses Mamm Genome 2007 18 9 635 645 10.1007/s00335-007-9046-0 17694346

[B62] Mehrabian M Allayee H Stockton J Lum PY Drake TA Castellani LW Suh M Armour C Edwards S Lamb J Lusis AJ Schadt EE Integrating genotypic and expression data in a segregating mouse population to identify 5-lipoxygenase as a susceptibility gene for obesity and bone traits Nat Genet 2005 37 11 1224 1233 10.1038/ng1619 16200066

[B63] Frazer KA Eskin E Kang HM Bogue MA Hinds DA Beilharz EJ Gupta RV Montgomery J Morenzoni MM Nilsen GB Pethiyagoda CL Stuve LL Johnson FM Daly MJ Wade CM Cox DR A sequence-based variation map of 8.27 million SNPs in inbred mouse strains Nature 2007 448 7157 1050 1053 10.1038/nature06067 17660834

[B64] Szatkiewicz JP Beane GL Ding Y Hutchins L Pardo-Manuel de Villena F Churchill GA An imputed genotype resource for the laboratory mouse Mamm Genome 2008 19 3 199 208 10.1007/s00335-008-9098-9 18301946 PMC2725522

[B65] Yang H Ding Y Hutchins LN Szatkiewicz J Bell TA Paigen BJ Graber JH de Villena FP Churchill GA A customized and versatile high-density genotyping array for the mouse Nat Methods 2009 6 9 663 666 10.1038/nmeth.1359 19668205 PMC2735580

[B66] Diament AL Warden CH Multiple linked mouse chromosome 7 loci influence body fat mass Int J Obes Relat Metab Disord 2004 28 2 199 210 14569280 10.1038/sj.ijo.0802516

[B67] Wallingford N Perroud B Gao Q Coppola A Gyengesi E Liu Z-W Gao X-B Diament A Haus KA Shariat-Madar Z Mahdi F Wardlaw SL Schmaier AH Warden CH Diano S Prolylcarboxypeptidase regulates food intake by inactivating α-MSH in rodents J Clin Invest 2009 119 8 2291 2303 19620781 10.1172/JCI37209 PMC2719925

[B68] Chen R Morgan AA Dudley J Deshpande T Li L Kodama K Chiang AP Butte AJ FitSNPs: highly differentially expressed genes are more likely to have variants associated with disease Genome Biol 2008 9 12 R170 10.1186/gb-2008-9-12-r170 19061490 PMC2646274

[B69] Gorlov IP Gallick GE Gorlova OY Amos C Logothetis CJ GWAS meets microarray: are the results of genome-wide association studies and gene-expression profiling consistent? Prostate cancer as an example PLoS ONE 2009 4 8 e6511 10.1371/journal.pone.0006511 19652704 PMC2714961

[B70] Kang HM Zaitlen NA Wade CM Kirby A Heckerman D Daly MJ Eskin E Efficient control of population structure in model organism association mapping Genetics 2008 178 3 1709 1723 10.1534/genetics.107.080101 18385116 PMC2278096

[B71] Pritchard JK Przeworski M Linkage disequilibrium in humans: models and data Am J Hum Genet 2001 69 1 1 14 10.1086/321275 11410837 PMC1226024

[B72] Idaghdour Y Storey JD Jadallah SJ Gibson G A genome-wide gene expression signature of environmental geography in leukocytes of Moroccan Amazighs PLoS Genet 2008 4 4 e1000052 10.1371/journal.pgen.1000052 18404217 PMC2290968

[B73] Stranger BE Nica AC Forrest MS Dimas A Bird CP Beazley C Ingle CE Dunning M Flicek P Koller D Montgomery S Tavaré S Deloukas P Dermitzakis ET Population genomics of human gene expression Nat Genet 2007 39 10 1217 1224 10.1038/ng2142 17873874 PMC2683249

[B74] Yang H Harrington CA Vartanian K Coldren CD Hall R Churchill GA Randomization in laboratory procedure is key to obtaining reproducible microarray results PLoS ONE 2008 3 11 e3724 10.1371/journal.pone.0003724 19009020 PMC2579585

[B75] Yang H Bell TA Churchill GA Pardo-Manuel de Villena F On the subspecific origin of the laboratory mouse Nat Genet 2007 39 9 1100 1107 10.1038/ng2087 17660819

[B76] Churchill GA Airey DC Allayee H Angel JM Attie AD Beatty J Beavis WD Belknap JK Bennett B Berrettini W Bleich A Bogue M Broman KW Buck KJ Buckler E Burmeister M Chesler EJ Cheverud JM Clapcote S Cook MN Cox RD Crabbe JC Crusio WE Darvasi A Deschepper CF Doerge RW Farber CR Forejt J Gaile D Garlow SJ The Collaborative Cross, a community resource for the genetic analysis of complex traits Nat Genet 2004 36 11 1133 1137 10.1038/ng1104-1133 15514660

[B77] Karp CL Grupe A Schadt E Ewart SL Keane-Moore M Cuomo PJ Kohl J Wahl L Kuperman D Germer S Aud D Peltz G Wills-Karp M Identification of complement factor 5 as a susceptibility locus for experimental allergic asthma Nat Immunol 2000 1 3 221 226 10.1038/79759 10973279

[B78] PubMed http://www.ncbi.nlm.nih.gov/sites/entrez?db=PubMed

[B79] UCSC Genome Browser http://genome.ucsc.edu

[B80] Shifman S Bell JT Copley RR Taylor MS Williams RW Mott R Flint J A high-resolution single nucleotide polymorphism genetic map of the mouse genome PLoS Biol 2006 4 12 e395 10.1371/journal.pbio.0040395 17105354 PMC1635748

[B81] Ensembl Genome Browser http://www.ensembl.org

[B82] Curwen V Eyras E Andrews TD Clarke L Mongin E Searle SMJ Clamp M The Ensembl Automatic Gene Annotation System Genome Res 2004 14 5 942 950 10.1101/gr.1858004 15123590 PMC479124

[B83] Bradford GE Famula TR Evidence for a major gene for rapid postweaning growth in mice Genet Res 1984 44 3 293 308 10.1017/S0016672300026537 6530139

[B84] Horvat S Medrano JF Lack of Socs2 expression causes the high-growth phenotype in mice Genomics 2001 72 2 209 212 10.1006/geno.2000.6441 11401434

[B85] American Association for Accreditation of Laboratory Animal Care http://www.aaalac.org

[B86] R Development Core Team R: A language and environment for statistical computing 2005 Vienna, Austria: R Foundation for Statistical Computing

[B87] Hsu F Kent WJ Clawson H Kuhn RM Diekhans M Haussler D The UCSC Known Genes Bioinformatics (Oxford, England) 2006 22 9 1036 1046 10.1093/bioinformatics/btl048 16500937

[B88] Kent WJ BLAT--the BLAST-like alignment tool Genome Res 2002 12 4 656 664 11932250 10.1101/gr.229202 PMC187518

[B89] RefSeq genomic coordinates (Build37) ftp://ftp.ncbi.nlm.nih.gov/genomes/M_musculus/mapview/

[B90] Bioconductor http://www.bioconductor.org

[B91] Wu H Kerr M Cui X Churchill G Parmigiani G Garett ES Irizarry RA Zeger SL MAANOVA: A Software Package for the Analysis of Spotted cDNA Microarray Experiments The analysis of gene expression data: methods and software 2002 New York: Springer

[B92] Cui X Hwang JT Qiu J Blades NJ Churchill GA Improved statistical tests for differential gene expression by shrinking variance components estimates Biostatistics 2005 6 1 59 75 10.1093/biostatistics/kxh018 15618528

[B93] Yekutieli D Benjamini Y The control of the FDR multiple testing under dependency Ann Stat 2001 29 4 1165 1188 10.1214/aos/1013699998

[B94] CGD Strain Comparison http://cgd.jax.org/straincomparison

[B95] Ways JA Cicila GT Garrett MR Koch LG A genome scan for Loci associated with aerobic running capacity in rats Genomics 2002 80 1 13 20 10.1006/geno.2002.6797 12079278

[B96] Koudande OD van Arendonk JA Iraqi F Marker-assisted introgression of trypanotolerance QTL in mice Mamm Genome 2005 16 2 112 119 10.1007/s00335-004-2314-3 15859356

[B97] Carr LG Kimpel MW Liang T McClintick JN McCall K Morse M Edenberg HJ Identification of candidate genes for alcohol preference by expression profiling of congenic rat strains Alcohol Clin Exp Res 2007 31 7 1089 1098 10.1111/j.1530-0277.2007.00397.x 17451403 PMC4455872

[B98] Yagil C Sapojnikov M Kreutz R Katni G Lindpaintner K Ganten D Yagil Y Salt susceptibility maps to chromosomes 1 and 17 with sex specificity in the Sabra rat model of hypertension Hypertension 1998 31 1 119 124 9449402 10.1161/01.hyp.31.1.119

[B99] Meng H Garrett MR Dene H Rapp JP Localization of a blood pressure QTL to a 2.4-cM interval on rat chromosome 9 using congenic strains Genomics 2003 81 2 210 220 10.1016/S0888-7543(03)00003-X 12620399

[B100] Johannesson M Olsson LM Lindqvist AK Moller S Koczan D Wester-Rosenlof L Thiesen HJ Ibrahim S Holmdahl R Gene expression profiling of arthritis using a QTL chip reveals a complex gene regulation of the Cia5 region in mice Genes Immunol 2005 6 7 575 583 10.1038/sj.gene.6364242 16015370

[B101] Chiu S Kim K Haus KA Espinal GM Millon LV Warden CH Identification of positional candidate genes for body weight and adiposity in subcongenic mice Physiol Genomics 2007 31 1 75 85 10.1152/physiolgenomics.00267.2006 17536020

[B102] Kirstein SL Davidson KL Ehringer MA Sikela JM Erwin VG Tabakoff B Quantitative trait loci affecting initial sensitivity and acute functional tolerance to ethanol-induced ataxia and brain cAMP signaling in BXD recombinant inbred mice J Pharmacol Exp Ther 2002 302 3 1238 1245 10.1124/jpet.302.3.1238 12183685

[B103] Kumar KG Richards BKS Transcriptional profiling of chromosome 17 QTL for carbohydrate and total calorie intake in a mouse congenic strain reveals candidate genes and pathways J Nutrigenet Nutrigenomics 2008 1 4 155 171 10.1159/000113657 PMC2745168 19776624

[B104] Bennett B Beeson M Gordon L Carosone-Link P Johnson TE Genetic dissection of quantitative trait loci specifying sedative/hypnotic sensitivity to ethanol: mapping with interval-specific congenic recombinant lines Alcoholism ClinExper Res 2002 26 11 1615 1624 10.1097/01.ALC.0000037136.49550.B3 12436049

[B105] de Ledesma AM Desai AN Bolivar VJ Symula DJ Flaherty L Two new behavioral QTLs, Emo4 and Reb1, map to mouse Chromosome 1: Congenic strains and candidate gene identification studies Mamm Genome 2006 17 2 111 118 10.1007/s00335-005-0107-y 16465591

[B106] Letwin NE Kafkafi N Benjamini Y Mayo C Frank BC Luu T Lee NH Elmer GI Combined application of behavior genetics and microarray analysis to identify regional expression themes and gene-behavior associations J Neurosci 2006 26 20 5277 5287 10.1523/JNEUROSCI.4602-05.2006 16707780 PMC6675305

[B107] Yu X Bauer K Wernhoff P Koczan D Moller S Thiesen HJ Ibrahim SM Fine mapping of collagen-induced arthritis quantitative trait loci in an advanced intercross line J Immunol 2006 177 10 7042 7049 17082620 10.4049/jimmunol.177.10.7042

[B108] Dyck PA Hoda F Osmer ES Green RM Microarray analysis of hepatic gene expression in gallstone-susceptible and gallstone-resistant mice Mamm Genome 2003 14 9 601 610 10.1007/s00335-003-2269-9 14629110

[B109] Paigen B Schork NJ Svenson KL Cheah YC Mu JL Lammert F Wang DQ Bouchard G Carey MC Quantitative trait loci mapping for cholesterol gallstones in AKR/J and C57L/J strains of mice Physiol Genomics 2000 4 1 59 65 11074014 10.1152/physiolgenomics.2000.4.1.59

[B110] Mahler M Most C Schmidtke S Sundberg JP Li R Hedrich HJ Churchill GA Genetics of colitis susceptibility in IL-10-deficient mice: backcross versus F2 results contrasted by principal component analysis Genomics 2002 80 3 274 282 10.1006/geno.2002.6840 12213197

[B111] Stylianou IM Clinton M Keightley PD Pritchard C Tymowska-Lalanne Z Bunger L Horvat S Microarray gene expression analysis of the Fob3b obesity QTL identifies positional candidate gene Sqle and perturbed cholesterol and glycolysis pathways Physiol Genomics 2005 20 3 224 232 15598878 10.1152/physiolgenomics.00183.2004

[B112] Farahani P Chiu S Bowlus CL Boffelli D Lee E Fisler JS Krauss RM Warden CH Obesity in BSB mice is correlated with expression of genes for iron homeostasis and leptin Obes Res 2004 12 2 191 204 10.1038/oby.2004.26 14981211

[B113] Beamer WG Shultz KL Churchill GA Frankel WN Baylink DJ Rosen CJ Donahue LR Quantitative trait loci for bone density in C57BL/6J and CAST/EiJ inbred mice Mamm Genome 1999 10 11 1043 1049 10.1007/s003359901159 10556421

[B114] Ganguly K Stoeger T Wesselkamper SC Reinhard C Sartor MA Medvedoic M Tomlinson CR Bolle I Mason JM Leikauf GD Schulz H Candidate genes controlling pulmonary function in mice: transcript profiling and predicted protein structure Physiol Genomics 2007 31 410 21 10.1152/physiolgenomics.00260.2006 17804602

[B115] Fisher P Hedeler C Wolstencroft K Hulme H Noyes H Kemp S Stevens R Brass A A systematic strategy for large-scale analysis of genotype phenotype correlations: identification of candidate genes involved in African trypanosomiasis Nucleic Acids Res 2007 35 16 5625 5633 10.1093/nar/gkm623 17709344 PMC2018629

[B116] Eaves IA Wicker LS Ghandour G Lyons PA Peterson LB Todd JA Glynne RJ Combining mouse congenic strains and microarray gene expression analyses to study a complex trait: the NOD model of type 1 diabetes Genome Res 2002 12 2 232 243 10.1101/gr.214102. Article published online before print in January 2002 11827943

