# Supplementary material for: Serious limitations of the QTL/Microarray approach for QTL gene discovery
Source: BMC Biol. 2010 Jul 12;8:96. doi: 10.1186/1741-7007-8-96 (PMC2919467; doi:10.1186/1741-7007-8-96)
Supplement: Additional file 2 — List of phenotypes measured in three congenic strains HG2D, HG11, HG17. The file contains a list, symbol and units of measurement for 16 phenotypes measured in the mice. [file 1741-7007-8-96-S2.PDF]

Additional File 2: List of phenotypes measured in three congenic strains HG2D, HG11, HG17. List, symbols and units of measurement for 16 phenotypes measured in the mice.

Symbol	Phenotype	Unit
BW	Body weight at 2, 3, 6, or 9 wk	g
SPL	Spleen weight	mg
KID	Right and left kidney weight	mg
LIV	Liver weight	g
HRT	Heart weight	g
MUS	Gastrocnemius muscle weight	g
BRN	Brain weight	mg
GFP	Gonadal white adipose weight	mg
FFP	Femoral white adipose weight	mg
RFP	Retroperitoneal white adipose weight	mg
MFP	Mesenteric white adipose weight	mg
FAT	GFP + FFP + RFP + MFP	g
LBW	Lean body weight (9WK-FAT)	g
TAIL	Tail length	cm
NA	Nasal-anus length	cm
FEM	Femur length	mm
