# Supplementary material for: Serious limitations of the QTL/Microarray approach for QTL gene discovery
Source: BMC Biol. 2010 Jul 12;8:96. doi: 10.1186/1741-7007-8-96 (PMC2919467; doi:10.1186/1741-7007-8-96)
Supplement: Additional file 3 — QTL located within the limits of the donor regions for the HG2D, HG11, and HG17 congenic strains. The file contains a table with interval limits representing a non-redundant set of QTL from the cited references at the highest resolution currently known. [file 1741-7007-8-96-S3.PDF]

Additional File 3. QTLs located within the limits of the donor regions for the HG2D, HG11, and HG17 congenic strains. These interval limits represent a non-redundant set of QTLs from the cited references at the highest resolution currently known. Column 'Type' indicates QTL's mode of action: F=females only, M=males only, MF=Sex independent, I=sex antagonistic.

Name	Trait	Type	Chr	Marker1	Marker2	Mb1	Mb2	Reference
Fatq1	FAT (g)	F	2	D2Mit207	D2Mit260	112.0	149.0	[1]
Fatq2	FAT (g)	M	2	D2Mit262	D2Mit148	155.7	178.5	[1]
Swq6	SPL (mg)	FM	2	D2Mit260	D2Mit456	149.0	168.8	[1]
Kwq7	KID (mg)	M	2	D2Mit389	D2Mit17	103.1	122.6	[1]
Kwq8	KID (mg)	FM	2	D2Mit454	D2Mit213	164.1	174.4	[1]
Tailq7	TAIL (cm)	FM	2	D2Mit160	D2Mit212	84.8	141.8	[1]
Bdlnq7	NA (cm)	FM	2	D2Mit93	D2Mit454	76.7	164.1	[1]
Tcq1	TC (mg/dliter)	FM	2	D2Mit207	D2Mit224	112.0	129.1	[1]
Tcq2	TC (mg/dliter)	FM	2	D2Mit17	D2Mit456	122.6	168.8	[1]
Wg7	BW (g)	I	11	D11Mit261	D11Mit224	62.0	108.3	[1]
Lwq9	LIV (g)	M	11	D11Mit280	D11Mit67	76.9	96.8	[1]
Kwq9	KID (mg)	FM	11	D11Mit280	D11Mit67	76.9	96.8	[1]
Brwq1	BRN (mg)	FM	11	D11Mit280	D11Mit10	76.9	104.5	[1]
Tailq8	TAIL (cm)	M	11	D11Mit36	D11Mit255	83.6	114.1	[1]
Gfpq1	GFP (mg)	MF	11	D11Mit261	D11Mit255	62.0	114.1	[1]
Feml4	FEM (mm)	F	11	D11Mit5	D11Mit36	67.0	83.6	[1]
Wg2a	BW (g)	MF	2	D2Ucd15	D2Mit94	74.8	80.0	[2]
Wg2b	BW + FAT (g)	MF	2	D2Mit476	D2Mit420	86.3	118.2	[2]
Wg2c	FAT (g)	MF	2	D2Mit420	D2Mit107	118.2	133.1	[2]
Wg2d	FAT (g)	MF	2	D2Mit194	D2Mit196	143.8	160.3	[2]
Fem17q1	FEM (mm)	MF	17	rs33779311	rs13483066	25.2	63.7	[3]
Wg17q3	BW (g)	MF	17	rs29663729	rs13483066	55.2	63.7	[3]
Wg17q5	LBW (g)	MF	17	D17Mit6	rs29663747	52.7	60.7	[3]
Adip17q1	FAT (g)	MF	17	rs33684649	rs13464868	7.5	15.6	[3]
Livw17q1	LIV (g)	MF	17	D17Mit196	D17Mit118	5.9	64.8	[3]

References

1. Farber CR, Medrano JF: **Fine mapping reveals sex bias in quantitative trait loci affecting growth, skeletal size and obesity-related traits on mouse chromosomes 2 and 11**. *Genetics* 2007, **175**(1):349-360.
2. Farber CR, Medrano JF: **Dissection of a genetically complex cluster of growth and obesity QTLs on mouse chromosome 2 using subcongenic intercrosses**. *Mamm Genome* 2007, **18**(9):635-645.
3. Verdugo RA, Farber CR, Medrano JF: **Fine mapping of quantitative trait loci chromosome 17 for body mass and obesity in the mouse.** *Unpublished results.*
